# Spinal Muscular Atrophy in the Black South African Population: A Matter of Rearrangement?

**DOI:** 10.3389/fgene.2020.00054

**Published:** 2020-02-13

**Authors:** Elana Vorster, Fahmida B. Essop, John L. Rodda, Amanda Krause

**Affiliations:** ^1^National Health Laboratory Service and School of Pathology, University of the Witwatersrand, Johannesburg, South Africa; ^2^Department of Paediatrics, University of the Witwatersrand, Johannesburg, South Africa

**Keywords:** spinal muscular atrophy, survival motor neuron 1, survival motor neuron 2, multiplex ligation-dependent probe amplification, copy number variations, rearrangement, South Africa

## Abstract

Spinal muscular atrophy (SMA) is a neuromuscular disorder, characterized by muscle atrophy and impaired mobility. A homozygous deletion of survival motor neuron 1 (*SMN1*), exon 7 is the main cause of SMA in ~94% of patients worldwide, but only accounts for 51% of South African (SA) black patients. *SMN1* and its highly homologous centromeric copy, survival motor neuron 2 (*SMN2)*, are located in a complex duplicated region. Unusual copy number variations (CNVs) have been reported in black patients, suggesting the presence of complex pathogenic rearrangements. The aim of this study was to further investigate the genetic cause of SMA in the black SA population. Multiplex ligation-dependent probe amplification (MLPA) testing was performed on 197 unrelated black patients referred for SMA testing (75 with a homozygous deletion of *SMN1*, exon 7; 50 with a homozygous deletion of *SMN2*, exon 7; and 72 clinically suggestive patients with no homozygous deletions). Furthermore, 122 black negative controls were tested. For comparison, 68 white individuals (30 with a homozygous deletion of *SMN1*, exon 7; 8 with a homozygous deletion of *SMN2*, exon 7 and 30 negative controls) were tested. Multiple copies (>2) of *SMN1*, exon 7 were observed in 50.8% (62/122) of black negative controls which could mask heterozygous *SMN1* deletions and potential pathogenic CNVs. MLPA is not a reliable technique for detecting carriers in the black SA population. Large deletions extending into the rest of *SMN1* and neighboring genes were more frequently observed in black patients with homozygous *SMN1*, exon 7 deletions when compared to white patients. Homozygous *SMN2*, exon 7 deletions were commonly observed in black individuals. No clear pathogenic CNVs were identified in black patients but discordant copy numbers of exons suggest complex rearrangements, which may potentially interrupt the *SMN1* gene. Only 8.3% (6/72) of clinically suggestive patients had heterozygous deletions of *SMN1*, exon 7 (1:0) which is lower than previous SA reports of 69.5%. This study emphasizes the lack of understanding of the architecture of the *SMN* region as well as the cause of SMA in the black SA population. These factors need to be taken into account when counseling and performing diagnostic testing in black populations.

## Introduction

Spinal muscular atrophy (SMA) is an autosomal recessive neurological disorder, characterized by the progressive degeneration of anterior horn cells (lower motor neurons) of the spinal cord, causing symmetrical muscle atrophy, weakness and paralysis. Historically, SMA was categorized into four clinical subtypes (SMA I–IV), ranging in severity, maximum muscle activity achieved, and age of onset, although it has been suggested that the SMA phenotype rather spans a continuum (Prior et al., 2004). SMA type I is the most severe form, with onset usually at birth or before six months with an average lifespan of two years. SMA type II is an intermediate form with an onset between 6 and 18 months (Fried and Emery, 1971); SMA type III is a mild form with onset after 18 months (Kugelberg and Welander, 1956) and SMA type IV is the mildest form with adult onset (Pearn et al., 1978).

A previous study suggested that the clinical presentation of SMA in black South African (SA) patients differs from worldwide reports with more frequent involvement of facial muscles in the severe infantile form of SMA leading to an expressionless facies (Moosa and Dawood, 1990). This is supported by clinical observation, but has not been scientifically documented.

SMA has been reported to be the second most common autosomal recessive disorder in Caucasian individuals after cystic fibrosis. The predicted birth incidence of SMA varies between 1 in 6,000 and 1 in 10,000 with a carrier frequency estimated at 1 in 40 to 1 in 60 worldwide (Hendrickson et al., 2009). The birth incidence of SMA in black SA patients has been estimated to be much higher at 1 in 3,574. This indicates that SMA may have a higher birth incidence than albinism (birth incidence: 1 in 3,900) in the black SA population. The carrier rate of SMA was previously estimated to be 1 in 23 in the white SA population and 1 in 50 in the black SA population (Labrum et al., 2007).

SMA is caused by mutations within the survival motor neuron 1 gene (*SMN1*; OMIM #600354^1^). A homozygous deletion of *SMN1*, exon 7 is reported to cause SMA in ~94% of patients with SMA worldwide (Hendrickson et al., 2009). In contrast, only 51% of SA SMA cases have been reported to be caused by a homozygous deletion of *SMN1*, exon 7 (Stevens et al., 1999; Labrum et al., 2007). An *SMN1* deletion in conjunction with a second mutation, results in a compound heterozygote pattern and accounts for an additional 2–5% of patients with SMA worldwide (Wirth, 2000). The heterozygous deletion of *SMN1*, exon 7 rate in black SA patients with SMA who tested negative for the homozygous *SMN1*, exon 7 deletion, was previously reported to be as high at 69.5%, supporting the diagnosis of SMA in these patients and suggesting that SMA is probably due to additional unidentified mutations in this region (Labrum et al., 2007).

The *SMN1* gene and its highly homologous copy, survival motor neuron 2 (*SMN2;* OMIM #601627^1^) are located in the *SMN* region on chromosome 5q13. The *SMN* region consists of multiple copy genes, pseudogenes (Selig et al., 1995), repetitive sequences (Bürglen et al., 1996), and retrotransposon-like elements (Francis et al., 1995), resulting in a large 500 kb inverted duplication, containing both a telomeric copy (*SMN1*) and a centromeric copy (*SMN2*) of the region. The historic terms, “telomeric” and “centromeric” refer to the relative positions of the *SMN1* and *SMN2* genes, respectively, within the *SMN* critical region at chromosome 5q13. As a result of the complexity and hypervariability of the region, there is no current complete and accurate map of the *SMN* region.

Homozygous deletions of the centromeric *SMN2*, exon 7 are not thought to be pathogenic (Schwartz et al., 1997), but are commonly encountered in black SA patients referred for SMA testing (Stevens et al., 1999; Labrum et al., 2007). A number of studies have suggested that *SMN2* acts as a disease modifying gene as SMA disease severity is inversely correlated with *SMN2* copy number (McAndrew et al., 1997; Wirth et al., 1999; Feldkötter et al., 2002; Jedrzejowska et al., 2008). SMA type I patients tend to have two copies of *SMN2*, type II and type 3b (onset before three years)— three copies, SMA type IIIb (onset after three years)— four copies and SMA type IV— four to six copies (reviewed by Mercuri et al., 2018).

Recombination between *SMN1* and *SMN2* could potentially interrupt a critical region of *SMN1*, leading to the loss of full-length functional SMN transcripts. SMA type II and III patients have been shown to have gene conversions from *SMN1* to *SMN2* rather than deletions, resulting in a higher copy number of *SMN2*, which has been associated with a milder phenotype (Campbell et al., 1997). A high frequency (31.5%) of black SA patients with SMA were shown to have smaller deletions including *SMN1*, exon 7, but with exon 8 present, possibly due to gene conversions (Stevens et al., 1999). Additional evidence for this hypothesis was reported by Labrum et al. (2007) who observed a lower frequency of large deletions spanning *SMN1*, exons 7, 8, and the NLR family, apoptosis inhibitory protein gene (*NAIP*) in black SA patients (9.8%) when compared to white SA patients (41.7%).

Other genes located in the duplicated *SMN* region at chromosome 5q13 include *NAIP*, *GTF2H2*, and *SERF1A* and their multiple pseudo copies. The lack of understanding of the physical structure and orientation of these genes in the *SMN* region, hampers the better understanding of the role of these genes in the SMA disease mechanism.

The SMN protein is present in both the cytoplasm and nucleus of all cells, but is particularly abundant in motor neurons. The SMN protein's main function involves the assembly of small nuclear ribonucleoprotein (snRNP) complexes important for pre-messenger RNA splicing (Markowitz et al., 2012).

*SMN1* and *SMN2* differ in only 5 nucleotides of sequence, with the critical difference being a silent C to T transition at cDNA position 840 (c.840C > T) in *SMN2*, resulting in the exclusion of exon 7 during splicing and causing the majority of *SMN2* transcripts to be truncated and unstable (Lorson et al., 1999; Monani et al., 1999). Only 20% of the total full-length *SMN* (*FL-SMN*) transcript is produced from the *SMN2* gene, which partly compensates for the lack of *FL-SMN* transcript produced from *SMN1* in patients with SMA, but does not produce sufficient SMN protein levels in motor neurons for their survival (Zheleznyakova et al., 2011). A milder phenotype (SMA types III and IV) have been associated with four or more copies of *SMN2* (Wirth et al., 2006). Recently, Nusinersen, an antisense oligonucleotide drug that modifies splicing of *SMN2*, has been shown to lead to an increase in the total *FL-SMN* transcripts of *SMN2*, leading to improvements in motor function (Finkel et al., 2017).

Approximately 4% of American and Canadian individuals have been found to have heterozygous *SMN1* deletions with two *SMN1* gene copies on a single chromosome in addition to a chromosome with a deletion of the *SMN1* gene (2:0 genotype) (Scheffer et al., 2001). These individuals are SMA carriers since they have the ability to pass on a deletion chromosome to subsequent generations. Carrier testing is compromised since quantitative techniques cannot distinguish between two copies in cis or trans of *SMN1*, one copy present on each chromosome or two copies of *SMN1* present on a single chromosome in conjunction with 0 copies on the second chromosome (McAndrew et al., 1997). It is recommended that potential carriers with multiple copies of *SMN1* need to be analyzed in a family context to try and clarify the phase of these copy number variations (CNVs) and to accurately assign carrier status.

Studies performed on various American population groups, showed an unusually high frequency of multiple copies of *SMN1* in the African American population when compared to other populations (Hendrickson et al., 2009; Sugarman et al., 2012). A study performed on unaffected individuals from various sub-Saharan African populations (Kenyan, Malian and Nigerian) confirmed this observation and showed a higher frequency of multiple copies of *SMN1* and deletions of *SMN2* than European populations (Sangaré et al., 2014).

SMA was previously thought to be rare in African populations with limited studies performed in Northern Africa (Tunisia, Egypt, Nigeria, Algeria and Senegal), but this was likely due to an underestimation (Pelleboer et al., 1989; Tazir and Geronimi, 1990; Shawky et al., 2001; Ndiaye et al., 2002; Mrad et al., 2006).

It has been hypothesized that complex population-specific rearrangements of the *SMN* region could cause SMA in the black SA population (Labrum et al., 2007; Vorster et al., 2011). The main aim of this study was to investigate CNVs of the *SMN* region using the P021 multiplex ligation-dependent probe amplification (MLPA) probe mix (MRC Holland, Amsterdam, Netherlands), which has multiple probes spanning the *SMN* region, in an attempt to identify potential pathogenic CNVs which could contribute to the disease mechanism of SMA in the black SA population. A better understanding of potential pathogenic CNVs of the *SMN* region could improve diagnostic testing for the 49% of black SA patients affected with SMA who currently test negative for the common homozygous *SMN1*, exon 7 deletion.

## Subjects, Materials, and Methods

### Subjects: U/U^b^ Patients

U/U^b^ patients (Unidentified mutation/Unidentified mutation genotype) represent black patients who presented with symptoms clinically suggestive of SMA and who previously tested negative for a homozygous deletion of *SMN1*, exon 7 in a diagnostic setting using an in-house PCR and restriction enzyme assay. U/U^b^ patients were identified and selected in collaboration with the Clinical Section of the Division of Human Genetics, molecular diagnostic laboratory, National Health Laboratory Service Johannesburg (NHLS), and the University of the Witwatersrand (WITS), henceforth referred to as “the Division” and in collaboration with the Departments of Paediatrics of the Chris Hani Baragwanath and Charlotte Maxeke Academic Hospitals.

In total, 72 U/U^b^ patients were identified, nine of whom had muscle biopsies suggestive of SMA. MLPA analysis was performed on these patients to identify potential pathogenic CNV patterns. DNA samples of family members of U/U^b^ patients were not available. These patients formed the main focus of this research study. DNA samples of all of these patients are stored in the Division.

### Groups Used for Comparison

#### N/N^b^ Individuals

N/N^b^ individuals (Negative/Negative genotype) represent black controls negative for SMA. MLPA analysis was performed on family members of 61 N/N^b^ families (200 individuals in total). In order to be included, DNA had to be available from two unrelated parents and at least one child. The unaffected parents of these families were used as negative controls in this study and consisted of a total of 122 unrelated N/N^b^ individuals. Haplotypes were constructed from the MLPA data and family pedigrees were drawn to investigate potential novel CNV events in these families.

#### N/N^w^ Individuals

N/N^w^ individuals (Negative/Negative genotype) represent white controls negative for SMA. To compare the typical non-pathogenic CNV patterns of black and white individuals, 30 random unrelated N/N^w^ individuals were tested on MLPA.

#### M_1_/M_1_^b^ Patients

M_1_/M_1_^b^ patients (Mutation 1: deletion of *SMN1*, exon 7/Mutation 1: deletion of *SMN1*, exon 7 genotype) represent black patients who were previously identified to have the common homozygous deletion of *SMN1*, exon 7 on a diagnostic PCR and restriction enzyme assay designed to detect and distinguish homozygous deletions of *SMN1*, exon 7 and *SMN2*, exon 7 (van der Steege et al., 1996). MLPA analysis was performed on 75 M_1_/M_1_^b^ patients to investigate the molecular structure of pathogenic CNV patterns, including the extent of homozygous deletions of *SMN1*, exon 7 and potential gene conversion events. Furthermore, 25 of these patients formed part of families (71 individuals in total). MLPA was performed on all family members and haplotypes were constructed from the MLPA data and family pedigrees to investigate the phase of potential common pathogenic CNV patterns.

#### M_1_/M_1_^w^ Patients

M_1_/M_1_^w^ patients (Mutation 1: deletion of *SMN1*, exon 7/Mutation 1: deletion of *SMN1*, exon 7 genotype) represent white patients who were previously identified to have a homozygous deletion of *SMN1*, exon 7 on the diagnostic PCR, and restriction enzyme assay. For comparison, 30 random unrelated M_1_/M_1_^w^ patients were tested to compare the molecular structure of pathogenic CNV patterns between M_1_/M_1_^b^ and M_1_/M_1_^w^ patients.

#### M_2_/M_2_^b^ Patients

M_2_/M_2_^b^ patients (Mutation 2: deletion of *SMN2*, exon 7/Mutation 2: deletion of *SMN2*, exon 7 genotype) represent black patients who were previously identified to have a homozygous deletion of *SMN2*, exon 7 on the diagnostic assay. Fifty M_2_/M_2_^b^ patients were tested on MLPA to determine the underlying molecular structure of this common CNV and to understand the interaction between the *SMN1* and *SMN2* genes.

#### M_2_/M_2_^w^ Patients

M_2_/M_2_^w^ patients (Mutation 2: deletion of *SMN2*, exon 7/Mutation 2: deletion of *SMN2*, exon 7 genotype) represent white patients who were previously identified to have a homozygous deletion of *SMN2*, exon 7 on the diagnostic assay. For comparison, eight random unrelated M_2_/M_2_^w^ patients were tested on MLPA to compare the molecular structure of this CNV between M_2_/M_2_^b^ and M_2_/M_2_^w^ patients. Only eight M_2_/M_2_^w^ patients were included in this group, since they were the only M_2_/M_2_^w^ patients available who have been identified in the white SA population.

### Methods

#### Dna Extraction

Genomic DNA was extracted from whole blood using the salting out method (Miller et al., 1988), a commercial DNA extraction kit (High Pure PCR Template Preparation Kit, Roche Diagnostics), or in the case of chorionic villus sampling (CVS) and amniocyte material, the phenol-chloroform extraction method was used (Barker, 2004). All samples were processed and extracted in a diagnostic setting, with stringent quality control. The P021 probe mix was validated on blood, amniocyte material, and CVS samples. A quantity of 50–250 ng of DNA is recommended for MLPA^2^. All DNA samples were normalized in order to accurately compare probe copy numbers to each other.

#### MLPA Analysis

The MLPA P021 probe mix (MRC Holland, Amsterdam, Netherlands) is mainly designed to detect *SMN1* and *SMN2*, exon 7 copy numbers. The P021 probe mix consists of a multiplex of 46 probes, consisting of seven DQ (dosage quality) control probes; two sex-chromosome specific probes (for gender determination and to detect sample mix-up); 22 internal reference probes, specific to various chromosomal regions and not associated with SMA; 15 probes specific to the SMN region (eight targeting the *SMN1* and *SMN2* genes and seven probes targeting neighboring genes). The DQ control probes amplify four Q fragments which determine whether sufficient DNA has been added to the reaction and whether ligation has been successful and two D fragments which determine whether successful denaturation of the DNA sample took place^2^.

Probes have been designed to target the critical one base pair difference between *SMN1* and *SMN2* in exon 7 and can therefore distinguish between exon 7 of *SMN1* and *SMN2*. Similarly, probes have been designed to target a one base pair difference between *SMN1* and *SMN2*, exon 8 and can therefore distinguish between exon 8 of *SMN1* and *SMN2*. Probes specific to exons 1, 4, and 6 of the *SMN1* and *SMN2* genes as well as neighboring genes in the *SMN* region have been included to assist with determining the extent of deletions (He et al., 2013). Neighbouring genes include the RAD17 checkpoint clamp loader component gene (*RAD17*) and telomeric as well as centromeric copies of the *NAIP* genes (*NAIP/NAIPΨ*), the general transcription factor IIH subunit 2 genes (*GTF2H2*), and the small EDRK-rich factor 1A genes (*SERF1A/1B*)^3^.

MLPA was performed using the Applied Biosystems (ABI) 9700 thermal cycler and fragment separation was performed using the ABI Genetic Analyzer 3130xl (Applied Biosystems, Foster City, CA, USA). Dosage analysis was performed using the freely available Coffalyser software (MRC Holland, Amsterdam, Netherlands) to quantify CNVs. By comparing the copy number of PCR products observed in a patient sample with endogenous reference probes and several external control samples, relative quantitative changes in DNA fragments can be determined (Schouten et al., 2002). The copy number of probe regions was determined using the parameters as set out in **Table 1**. MLPA results were analyzed by statistical analysis using Statistica (Dell, version 12.7) and Real Statistics Using Excel software^4^ to compare dosage trends between different patient groups and to determine significant differences among patient and control groups.

**Table 1:** The relationship of the P021 probe mix dosage quotient with copy number.

Dosage Quotient (DQ) distribution	Copy number of a single gene or region (e.g. *SMN1*, exon 7)	Copy number of two combined pseudogenes or regions (e.g. *SMN1/2*, exons 1, 4, 6 & 8)
0 « DQ < 0.35	0	0
0.35 « DQ < 0.65	1	2
0.65 « DQ < 1.35	2	4
1.35 « DQ < 1.65	3	6
1.65 « DQ < 2.35	4	8

Adapted from recommendations by MRC Holland (P021 SMA Product Description2).

MLPA has been shown to be a reliable technique to detect multiple copy numbers in regions of segmental duplication, without the need for repeat testing or replicates (Cantsilieris et al., 2014). Furthermore, the analytical sensitivity and specificity of the P021 probe mix has been reported to be >99%^2^. The P021 probe mix was validated using samples of individuals whose *SMN1* copy number was previously identified using the in-house PCR and restriction enzyme diagnostic assay or through ISO 17043 accredited external quality assessors, the European Molecular Genetics Quality Network (EMQN)^3^. Furthermore, negative, homozygous *SMN1*, exon 7 deletion and heterozygous *SMN1*, exon 7 deletion control samples were included in every experiment to ensure consistency among experiments. MLPA experiments were repeated and/or excluded when results did not adhere to quality requirements.

Haplotype analysis of pedigrees of N/N^b^ and M_1_/M_1_^b^ families were performed to determine common chromosomal CNV patterns/haplotypes in the black SA population. Multiple copies of genes in the *SMN* region were assumed to be located on chromosome 5 and the gene order was based on current map data from the Ensembl genome browser (genome built: GRCh38)^5^. Family pedigrees were categorized as informative if a clear pattern of inheritance from parents to their children could be established for each of the MLPA probes. Family pedigrees were categorized as uninformative if the phase of the CNVs could not be correctly determined. Multiple copies of a specific probe region complicate the assignment of phase due to various combinations being possible within a family and apparent discrepant results could arise due to potential novel CNV events, family members who are not related as specified or technical MLPA faults. All discrepant results were repeated on MLPA to ensure the accuracy of these results.

An ethics application was approved unconditionally by the WITS Medical Human Research Ethics Committee (ethics clearance number: M130950).

## Results

### MLPA Analysis

The results of MLPA analysis of *SMN1* and *SMN2* of the patient and control groups are summarized in **Table 2**. All MLPA data is available as **Supplementary Material**.

**Table 2:** Summary of telomeric *SMN1*, exons 7 and 8 and centromeric *SMN2*, exons 7 and 8 copy number across various patient and control groups.

Genotype	Definition of genotype	Copy number	Telomeric *SMN* region		Centromeric *SMN* region	
			*SMN1*, exon 7	*SMN1*, exon 8	*SMN2*, exon 7	*SMN2*, exon 8
**N/N^b^** **(n = 122)**	Negative black controls	0	0 (0%)	1 (0.8%)	15 (12.3%)	33 (27%)
		1	0 (0%)	0 (0%)	20 (16.4%)	55 (45.1%)
		2	60 (49.2%)	54 (44.3%)	73 (59.8%)	31 (25.4%)
		>2	62 (50.8%)	67 (54.9%)	14 (11.5%)	3 (2.5%)
**N/N^w^** **(n = 30)**	Negative white controls	0	0 (0%)	0 (0%)	2 (6.7%)	2 (6.7%)
		1	0 (0%)	0 (0%)	8 (26.6%)	12 (40%)
		2	29 (96.7%)	28 (93.3%)	18 (60%)	14 (46.6%)
		>2	1 (3.3%)	2 (6.7%)	2 (6.7%)	2 (6.7%)
**M_1_/M_1_^b^** **(n = 75)**	Black patients with homozygous *SMN1*, exon 7 deletion (Mutation 1)	0	75 (100%)	38 (50.7%)	0 (0%)	8 (10.7%)
		1	0 (0%)	22 (29.3%)	4 (5.3%)	15 (20%)
		2	0 (0%)	12 (16%)	63 (84%)	49 (65.3%)
		>2	0 (0%)	3 (4%)	8 (10.7%)	3 (4%)
**M_1_/M_1_^w^** **(n = 30)**	White patients with homozygous *SMN1*, exon 7 deletion (Mutation 1)	0	30 (100%)	24 (80%)	0 (0%)	0 (0%)
		1	0 (0%)	4 (13.3%)	2 (6.7%)	5 (16.7%)
		2	0 (0%)	2 (6.7%)	17 (56.7%)	13 (43.3%)
		>2	0 (0%)	0 (0%)	11 (36.7%)	12 (40%)
**M_2_/M_2_^b^** **(n = 50)**	Black patients with homozygous *SMN2*, exon 7 deletion (Mutation 2)	0	0 (0%)	0 (0%)	50 (100%)	49 (98%)
		1	2 (4%)	0 (0%)	0 (0%)	1 (2%)
		2	16 (32%)	21 (42%)	0 (0%)	0 (0%)
		>2	32 (64%)	29 (58%)	0 (0%)	0 (0%)
**M_2_/M_2_^w^** **(n = 8)**	White patients with homozygous *SMN2*, exon 7 deletion (Mutation 2)	0	0 (0%)	0 (0%)	8 (100%)	8 (100%)
		1	0 (0%)	0 (0%)	0 (0%)	0 (0%)
		2	5 (62.5%)	5 (62.5%)	0 (0%)	0 (0%)
		>2	3 (37.5%)	3 (37.5%)	0 (0%)	0 (0%)
**U/U^b^** **(n = 72)**	Black patients clinically suggestive of SMA and negative for a homozygous deletion of *SMN1*, exon 7 (unknown)	0	0 (0%)	0 (0%)	10 (13.9%)	17 (23.6%)
		1	6 (8.3%)	5 (6.9%)	8 (11.1%)	33 (45.8%)
		2	38 (52.8%)	31 (43.1%)	51 (70.8%)	19 (26.4%)
		>2	28 (38.9%)	36 (50%)	3 (4.2%)	3 (4.2%)

#### Comparative Analysis of N/N^b^ and N/N^w^ Individuals

**Figure 1:**
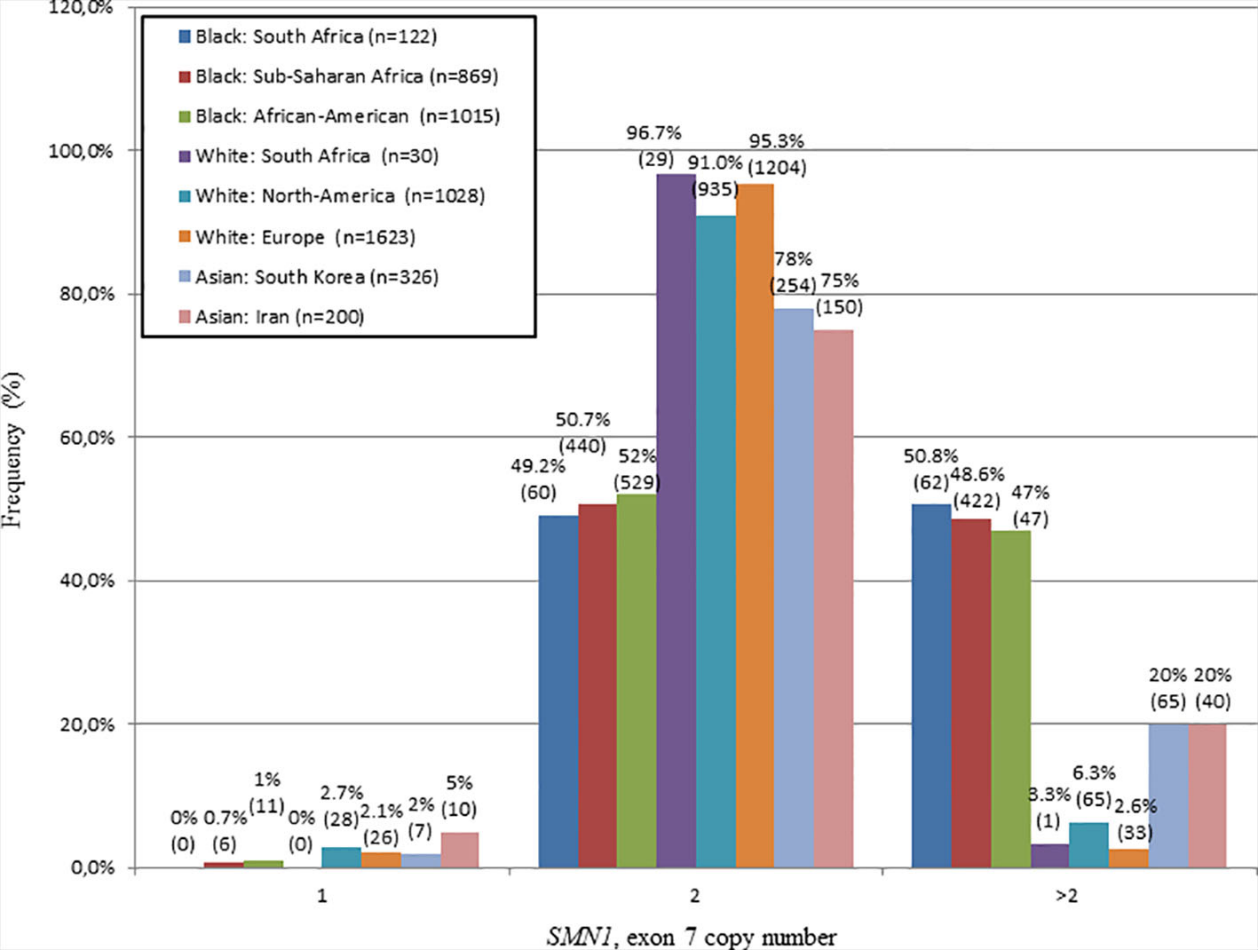
Comparison of the *SMN1*, exon 7 copy number distribution of N/N^b^ and N/N^w^ individuals with various international studies. Graph produced from combined data of this study and a study performed by Sangaré et al., 2014. A similar frequency of white individuals with 2 copies of *SMN1*, exon 7 was observed in SA, North-America and Europe. A similar frequency of black individuals with multiple copies (>2) of *SMN1*, exon 7 was observed in SA, Sub-Saharan Africa and North-America.

The *SMN1*, exon 7 copy number was found to differ statistically between N/N^b^ and N/N^w^ individuals (Kruskal-Wallis test: H = 32.7; p < 0.0001). In this study, 50.8% (62/122) of N/N^b^ individuals were found to have multiple copies (3–6) of *SMN1*, exon 7. These results stand in sharp contrast to trends observed in N/N^w^ individuals, with only 3.3% (1/30) with multiple copies of *SMN1*, exon 7. **Figure 1** compares the *SMN1* copy numbers observed in SA populations with various international population groups.

Similarly, the telomeric *SMN1*, exon 8 and *NAIP*, exon 5 copy numbers differed significantly between N/N^b^ and N/N^w^ individuals (Kruskal-Wallis test: H = 14.3; p = 0.0002 and H = 7.2; p = 0.0071, respectively). As seen with the *SMN1*, exon 7 region; 54.9% (67/122) of N/N^b^ individuals were found to have multiple copies (>2 copies) of *SMN1*, exon 8 and 37.7% (46/122) were found to have multiple copies of *NAIP*, exon 5, which are both assumed to co-locate with *SMN1* in the telomeric *SMN* region. Once again, these results stand in contrast to trends observed in N/N^w^ individuals, with only 6.7% (2/30) of individuals with multiple copies of *SMN1*, exon 8 and only 13.3% (4/30) with multiple copies of *NAIP*, exon 5. The *SMN1*, exon 7 and 8 copy numbers did not correlate fully, suggesting that some of these copies may not be contiguous.

The centromeric *SMN2*, exon 7 copy number was not found to differ significantly between N/N^b^ and N/N^w^ individuals (Kruskal-Wallis test: H = 0.09; p = 0.7652). The majority of N/N^b^ and N/N^w^ individuals had two copies of *SMN2*, exon 7; 59.8% (73/122) and 60% (18/30), respectively. The *SMN2*, exon 8 copy number was found to differ significantly between N/N^b^ and N/N^w^ individuals (Kruskal-Wallis test: H = 11.1; p = 0.0009). N/N^b^ individuals had a higher rate of homozygous *SMN2*, exon 8 deletions (27% (33/122) than N/N^w^ individuals (6.7% (2/30). This finding could be due to gene conversions from centromeric *SMN2*, exon 8 to telomeric *SMN1*, exon 8 resulting in hybrid genes consisting of *SMN1*, exon 7 and *SMN2*, exon 8. Deletions of these hybrid genes would result in a loss of *SMN1*, exon 7 in conjunction with *SMN2*, exon 8.

No N/N^b^ (0/122) and N/N^w^ (0/30) individuals appeared to have a detectable heterozygous deletion of *SMN1*, exon 7 (1:0 genotype), usually accepted to be carriers of SMA. Two N/N^b^ families had *de novo* CNV events with a new mutation rate of 3.3% (2/61).

**Figure 2:**
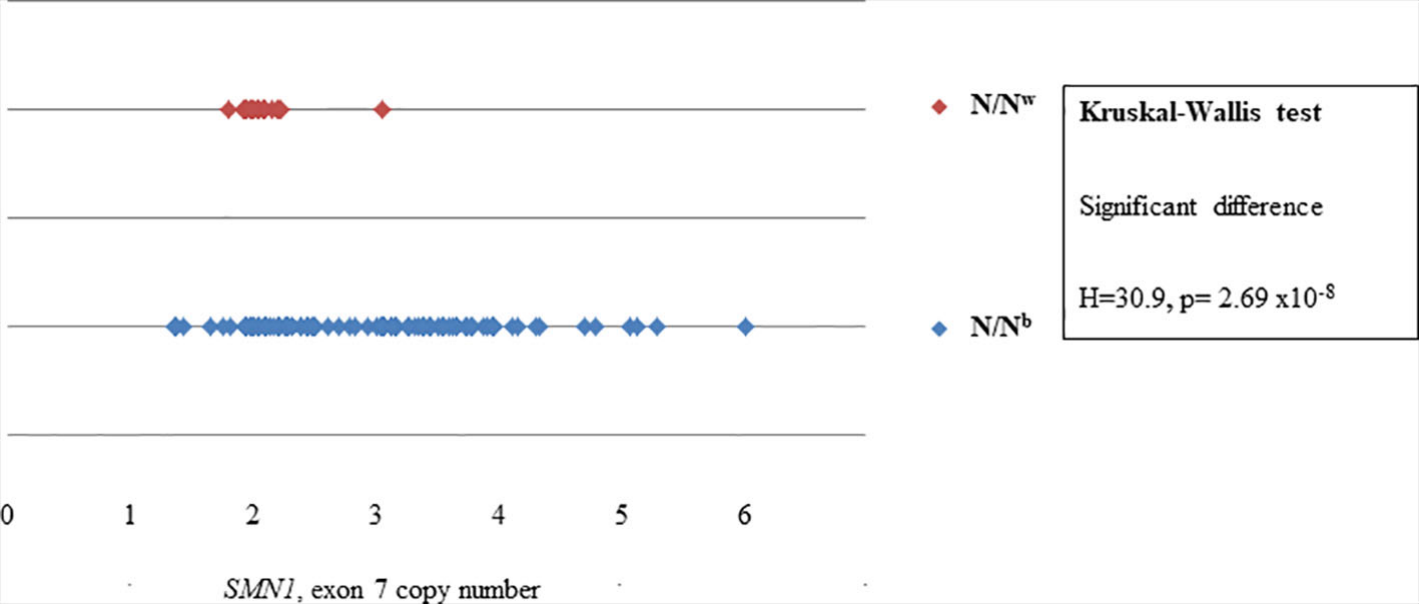
Scatterplot comparison of probe copy numbers of *SMN1*, exon 7 between N/N^b^ and N/N^w^ individuals. Whereas the copy number of *SMN1*, exon 7 of N/N^w^ individuals seem to cluster at 2 copies; the copy number of *SMN1*, exon 7 of N/N^b^ individuals varies extensively from 1 to 6.

N/N^b^ individuals had a significantly higher variance in copy number than N/N^w^ individuals for the majority of MLPA probes. Whereas the copy number of the telomeric *SMN1*, exons 7, 8 and *NAIP*, exon 5 of N/N^w^ individuals seem to cluster at two copies, as expected; the copy number of *SMN1*, exons 7, 8 and *NAIP*, exon 5 of N/N^b^ individuals varies extensively from 1 to 6. **Figure 2** shows the difference in variance in copy number of *SMN1*, exon 7 between N/N^b^ and N/N^w^ individuals. In N/N^w^ individuals the *SMN1*, exon 7 copy number clusters around integers (one or two copy numbers) whereas the *SMN1*, exon 7 copy number varies extensively in N/N^b^ individuals (one to six copy numbers). It is hypothesized that some of these multiple *SMN1* gene copies may be partial discontinuous gene copies which may be non-functional due to interruptions in the coding region.

Further, The *SERF1B* and *RAD17* copy numbers were not found to differ significantly between N/N^b^ and N/N^w^ individuals (Kruskal-Wallis test: H = 0.80; p = 0.367 and H = 0.00; p = 0.967, respectively). Multiple copies (more than 2) of *RAD17* were observed in 14.8% (18/122) of N/N^b^ individuals in contrast to no CNVs of *RAD17* in N/N^w^ individuals, suggesting that N/N^b^ individuals have a higher variability of these regions than N/N^w^ individuals.

#### Comparative Analysis of M_1_/M_1_^b^ and M_1_/M_1_^w^ Patients

As expected, MLPA analysis confirmed *SMN1*, exon 7 homozygous deletions in all 75 M_1_/M_1_^b^ patients and all 30 M_1_/M_1_^w^ patients. The telomeric *SMN1*, exon 8 and *NAIP*, exon 5 copy numbers were found to differ statistically between M_1_/M_1_^b^ and M_1_/M_1_^w^ patients (Kruskal-Wallis test: H = 7.8, p = 0.0053; H = 5.6, p = 0.0181, respectively). This significant difference could be due to homozygous *SMN1*, exon 8 deletions being more common in M_1_/M_1_^w^ patients [80% (24/30)] than M_1_/M_1_^b^ patients [50.7% (38/75)] and heterozygous deletions of *NAIP*, exon 5 being more common in M_1_/M_1_^w^ patients [56.7% (17/30)] than M_1_/M_1_^b^ patients [21.3% (16/75)].

The *SMN2*, exon 7 copy number was found to differ significantly between M_1_/M_1_^b^ and M_1_/M_1_^w^ patients (Kruskal-Wallis test: H = 9, p = 0.0027), likely due to a higher frequency of multiple copies (> 2) of this region in M_1_/M_1_^w^ patients [36.7% (11/30)] when compared to M_1_/M_1_^b^ patients [10.7% (8/75)]. Multiple copies of *SMN2*, exon 7 in conjunction with homozygous deletions of *SMN1*, exon 7 suggest gene conversion from telomeric *SMN1*, exon 7 to centromeric *SMN2*, exon 7 being more common in M_1_/M_1_^w^ patients.

Due to identical sequences of exons 1, 4, 6, and another region of exon 8 of the *SMN1* and *SMN2* genes, the P021 probe mix cannot distinguish between *SMN1* and *SMN2* for these regions and will give a combined copy number result (representing both the *SMN1* and *SMN2* genes: *SMN1/2*). An absence of these probes could therefore represent a deletion in either or both copies of the *SMN1* and *SMN2*, which complicates analysis.

A homozygous deletion of *SMN1/2*, exons 1, 4, and 6 were more frequently observed in M_1_/M_1_^b^ individuals [exon 1: 60% (45/75), exon 4: 61.3% (46/75) and exon 6: 62.7% (47/75) than M_1_/M_1_^w^ individuals (exon 1, 4 and 6: 31.3% (10/30)]. The *SMN1/2*, exons 4 and 6 copy numbers were found to differ statistically between M_1_/M_1_^b^ and M_1_/M_1_^w^ individuals (Kruskal-Wallis test: H = 4.4, p = 0.0355 and H = 4.2, p = 0.0399, respectively). The *SMN1/2*, exon 1 copy number was not found to differ significantly between M_1_/M_1_^b^ and M_1_/M_1_^w^ individuals (Kruskal-Wallis test: H = 1; p = 0.3188). The MLPA probes for exons 1, 4, and 6 cannot distinguish between *SMN1* and *SMN2* but since these patients have homozygous deletions of *SMN1*, exons 7 and 8, the exons 1, 4, and 6 deletions are most likely located in *SMN1*. The *SMN1/2*, exons 1, 4, and 6 copy numbers did not correlate fully with each other or with the *SMN1*, exon 7 and 8 copy numbers in M_1_/M_1_^b^ or M_1_/M_1_^w^ individuals suggesting that these copies may not be contiguous.

The *GTF2H2*, exon 5 and *NAIP/NAIPΨ*, exon 13 copy numbers were found to differ statistically between M_1_/M_1_^b^ and M_1_/M_1_^w^ individuals (Kruskal-Wallis test: H = 10.8, p = 0.001 and H = 13.8, p = 0.0002, respectively). A deletion of *GTF2H2*, exon 5 and *NAIP/NAIPΨ*, exon 13 were more frequently observed in M_1_/M_1_^b^ individuals [66.7% (50/75) and 61.3% (46/75) respectively] than M_1_/M_1_^w^ individuals [50% (15/30) and 23.3% (7/30) respectively].

*SERF1B* copy numbers were found to differ significantly between M_1_/M_1_^b^ and M_1_/M_1_^w^ individuals (Kruskal-Wallis test: H = 3.9, p = 0.0480), most likely due to a higher frequency of heterozygous deletions of *SERF1B* in 34.7% (26/75) of M_1_/M_1_^b^ individuals compared to 0% (0/30) in M_1_/M_1_^w^ individuals. There was no difference in copy number of *RAD17* between M_1_/M_1_^b^ and M_1_/M_1_^w^ individuals.

A higher frequency of deletions extending into the rest of *SMN1/2* (exons 1, 4, and 6), *NAIP/NAIPΨ*, exon 13, *GTF2H2*, exon 5 and *SERF1B* in M_1_/M_1_^b^ patients when compared to M_1_/M_1_^w^ patients suggest that large deletions are more common in M_1_/M_1_^b^ patients than M_1_/M_1_^w^ patients, discrepant from results obtained from *SMN1*, exon 8 and *NAIP*, exon 5 analysis.

#### Comparative Analysis of M_2_/M_2_^b^ and M_2_/M_2_^w^ Patients

From a previous retrospective audit of patients referred to the Division for SMA testing, performed from September 1991 to October 2015, it was shown that homozygous deletions of *SMN2*, exon 7 were identified in 12.4% (123/991) of black patients, 4.7% (9/192) of white patients, 4% (2/50) of Indian patients and 18.8% (3/16) of patients with mixed ancestry. There is a significantly higher percentage of *SMN2*, exon 7 deletions in black patients when compared to white patients (Chi-square test: χ^2^ = 11.64; p = 0.000645).

MLPA analysis confirmed homozygous *SMN2*, exon 7 deletions in 100% (50/50) of M_2_/M_2_^b^ patients and 100% (8/8) of M_2_/M_2_^w^ patients identified. Homozygous deletions of *SMN2*, exon 8 were detected in 98% (49/50) of M_2_/M_2_^b^ patients and 100% (8/8) of M_2_/M_2_^w^ patients. Only one M_2_/M_2_^b^ individual had a smaller deletion of *SMN2*, exon 7 which did not extend into exon 8. CNVs of *SMN2*, exons 7 and 8 were not found to differ statistically between M_2_/M_2_^b^ and M_2_/M_2_^w^ patients (Kruskal-Wallis test: H = 2.4; p = 0.1252 and H = 0.2; p = 0.6921, respectively).

CNVs of *NAIP/NAIPΨ*, exon 13 were found to differ statistically between M_2_/M_2_^b^ and M_2_/M_2_^w^ individuals (Kruskal-Wallis test: H = 5.6; p = 0.0177), most likely due to deletions being more frequently observed in M_2_/M_2_^b^ [44% (22/50) than M_2_/M_2_^w^ individuals (0% (0/8)].

CNVs of *SMN1/2*, exons 1 (Kruskal-Wallis test: H = 0.07; p = 0.7904), 4 (Kruskal-Wallis test: H = 2.5; p = 0.1107), and 6 (Kruskal-Wallis test: H = 0.8; p = 0.3579), *GTF2H2*, exon 5 (Kruskal-Wallis test: H = 1.98; p = 0.1596) and *SERF1B* (Kruskal-Wallis test: H = 0.02; p = 0.8855) were not found to differ statistically between M_2_/M_2_^b^ and M_2_/M_2_^w^ individuals. There was no difference in copy number of *RAD17* between M_2_/M_2_^b^ and M_2_/M_2_^w^ individuals. The MLPA probes for exons 1, 4, and 6 cannot distinguish between *SMN1* and *SMN2*, but since these patients have homozygous deletions of *SMN2*, exons 7 and 8, the exons 1, 4, and 6 deletions are most likely located in *SMN2*.

#### Comparative Analysis of U/U^b^ Patients With Black Control Groups (N/N^b^, M_1_/M_1_^b^ and M_2_/M_2_^b^)

Significant differences (p < 0.05) between U/U^b^ patients and M_1_M_1_^B^ and M_2_M_2_^B^ patients were observed, suggesting that hypothesized novel pathogenic CNVs are distinct from the common homozygous deletions of *SMN1*, exon 7 and *SMN2*, exon 7. U/U^b^ patients more closely resembled N/N^b^ individuals. Multiple copies (>2 copies) were observed for *SMN1*, exon 7 [38.9% (28/72) and *SMN1*, exon 8 [50% (36/72)] in U/U^b^ patients which is similar to that found in N/N^b^ individuals. A significant difference of the *SMN1*, exon 7 copy number between U/U^b^ patients and N/N^b^ individuals was observed and could be attributed to the presence of heterozygous *SMN1*, exon 7 deletions in 8.3% (6/72) of U/U^b^ patients, not observed in any N/N^b^ individuals [0% (0/122)]. This result stands in contrast to a previous South-African study that reported a heterozygous *SMN1*, exon 7 deletion rate of 69.5% (16/23) (Labrum et al., 2007).

### Haplotype and CNV Pattern Analysis N/N^b^ Families

Only 31.7% (19/60) of N/N^b^ families were completely informative where the phase of CNVs could be determined with certainty. For 60% (36/60) of N/N^b^ families, multiple combinations were possible, due to the presence of multiple copies of one or more probe regions. The exact locations of these multiple copies are uncertain. Discrepant results were observed in 5% (3/60) of N/N^b^ families possibly due to non-paternity or novel deletion/duplication events in the proband. Two N/N^b^ families had clear novel results with a new mutation rate of 3.3% (2/60). In total, 19 N/N^b^ families, consisting of 38 unrelated parents were found to be informative, from which 76 haplotypes were constructed. In total, 35 unique haplotypes were identified, emphasizing the high variability of this region.

#### M_1_/M_1_^b^ Families

Only 44% (11/25) of M_1_/M_1_^b^ families were completely informative where the phase of CNVs could be assigned with certainty. For 48% (12/25) of these M_1_/M_1_^b^ families, multiple combinations of CNVs were possible, due to the presence of multiple copies of one or more probes. Discrepant results were observed in 8% (2/25) of M_1_/M_1_^b^ families which could be due to a variety of causes such as non-paternity or novel deletion or duplication events in the proband. In total, 22 pathogenic haplotypes were constructed from probands from M_1_/M_1_^b^ families. Of these, 17 unique haplotypes were identified, once again emphasizing the high variability of this region.

## Discussion

The *SMN1* gene is the key gene associated with SMA with the SMN2 gene thought to have a disease-modifying effect. Current drug therapies are aimed at increasing the *FL-SMN* transcripts produced from *SMN2*. Potential large complex rearrangements of the *SMN* region may play a role in the SMA disease mechanism in the black SA population and may influence diagnosis and potentially the effect of drug therapies. Therefore it is valuable to investigate the genetic CNV background of the black SA population.

A major limitation of previous quantitative studies of the SMN region performed in African-American (Hendrickson et al., 2009; Sugarman et al., 2012) and sub-Saharan African populations (Sangaré et al., 2014) was that CNV analysis was performed in unaffected individuals, with the exception of prenatal screening performed by Sugarman *et al*. This study focuses on comparing CNVs in black individuals who are negative for SMA (N/N^b^) to identify non-pathogenic CNVs as well as patients with known homozygous *SMN1* and *SMN2*, exon 7 deletions (M_1_/M_1_^b^ and M_2_/M_2_^b^, respectively) and patients who are clinically suggestive of SMA (U/U^b^) to delineate potential pathogenic CNVs.

### Multiple Copies of the Telomeric Region (*SMN1*, Exons 7 and 8 and *NAIP*, Exon 5) Were Observed in N/N^b^ Individuals and Could Complicate Analysis

In this study, 50.8% of N/N^b^ individuals were found to have multiple (3–6) copies of *SMN1*, exon 7, which is similar to previous reports of 46.8% (Hendrickson et al., 2009) and 47.1% (Sugarman et al., 2012) in African-American individuals and a combined percentage of 48.6% in sub-Saharan African populations (Mali, Nigeria and Kenya) (adapted from Sangaré et al., 2014). In contrast, N/N^w^ individuals have a much lower percentage of multiple *SMN1*, exon 7 copies of 3.3%, which is comparable to previous reports of 6.3% in white North-American populations (Hendrickson et al., 2009) and a combined percentage of 2.6% in European populations (Germany, France and Sweden) (summarized in **Figure 1**, Feldkötter et al., 2002; Corcia et al., 2012).

Similarly, multiple copies of *SMN1*, exon 8 and *NAIP*, exon 5 were more frequently observed in N/N^b^ individuals (54.9% and 37.7%, respectively) when compared to N/N^w^ individuals (6.7% and 13.3%, respectively).

No SMA carriers (individuals with a heterozygous deletions of *SMN1*, exon 7) were identified in either N/N^b^ or N/N^w^ individuals in this study in contrast to the previously predicted SA carrier rate of 1/50 in the black population and 1/23 in the white population (Labrum et al., 2007). Small sample sizes could have caused this discrepancy in both N/N^b^ (n = 122) and N/N^w^ (n = 30) individuals in this study.

Further, the discrepancy in N/N^b^ individuals could also be due to two additional reasons. Firstly, MLPA analysis is a very robust technique, which has built-in statistical tests and extensive normalization to multiple exogenous regions, which are likely to yield more reliable and accurate results than the previously used in-house dosage system, which normalized results against a single exogenous region (Labrum et al., 2007). Secondly, it is likely that there is a higher frequency of heterozygous *SMN1* deletion carriers (2:0, 3:0, 4:0, 5:0, 6:0, etc.) in the black SA population, not detectable by either of these two assays. These results are supported by a previous study performed by Sugarman et al. (2012) who reported heterozygous *SMN1* deletions (2:0) to be more common in African-American individuals (27%, n = 4 883) when compared to white individuals (3.6%, n = 24 471). As a result, the carrier detection rate in African-American individuals was lower at 70% versus 91% in other population groups.

MLPA cannot provide information on the location or phase of multiple *SMN1*, exon 7 copies on an individual's chromosomes and therefore these multiple copies could be located on a single chromosome, resulting in a heterozygous *SMN1* deletion carrier profile. MLPA is therefore not a reliable technique to detect SMA carriers in the black SA population.

### Large Deletions Extending Into the Rest of the *SMN* Region Appear to Be More Common in M_1_/M_1_^b^ Patients

*SMN1*, exon 8 has been reported to be deleted together with *SMN1*, exon 7 in 93% of positive SMA cases (Lefebvre et al., 1995). Furthermore, *NAIP* deletions have been associated with *SMN1* deletions in 67% of SMA type I patients (Roy et al., 1995). A previous SA study proposed that M_1_/M_1_^w^ patients had larger homozygous deletions of *SMN1*, exon 7 also encompassing the telomeric *SMN1*, exon 8 and *NAIP* more often than M_1_/M_1_^b^ patients (Labrum et al., 2007). Furthermore, M_1_/M_1_^b^ patients were previously reported to have a homozygous *SMN1*, exon 7 deletion in conjunction with a homozygous *NAIP* deletion (*SMN1*, exon 8 is present), suggestive of a gene conversion from *SMN1*, exon 7 to *SMN2*, exon 7, more often than M_1_/M_1_^w^ patients (Stevens et al., 1999; Labrum et al., 2007). CNV results of *SMN1*, exon 7, 8 and *NAIP* from this study did not differ significantly from results from the previous study in either M_1_/M_1_^w^ (Chi-square test: χ^2^ = 0.9, p = 0.8) or M_1_/M_1_^b^ patients (Chi-square test: χ^2^ = 1.6, p = 0.7).

MLPA analysis using the P021 probe mix offers a more extensive look into the rest of the *SMN* region, suggesting a different hypothesis. *NAIP* and *GTF2H2* deletions have been observed in patients with SMA (Roy et al., 1995; Carter et al., 1997)) and could therefore provide some information on the extent of *SMN1*, exon 7 deletions. A higher frequency of homozygous and heterozygous deletions extending into the rest of *SMN1/2* (exons 1, 4, 6, and 8), *NAIP/NAIPΨ*, exon 13, *GTF2H2*, exon 5 and *SERF1B* were observed in M_1_/M_1_^b^ patients compared to M_1_/M_1_^w^ patients. These observations suggest that large deletions are more common in M_1_/M_1_^b^ patients than M_1_/M_1_^w^ patients contrasting results from only analyzing *SMN1*, exon 8 and *NAIP*, exon 5.

Hybrid genes could mask larger deletions of the *SMN1* gene and could have confounded previous SA reports (Stevens et al., 1999; Labrum et al., 2007), creating the impression of smaller deletions in M_1_/M_1_^b^ patients when compared to M_1_/M_1_^w^ patients. In support of this hypothesis, the CNVs of the centromeric *SMN2*, exons 7 and 8 do not correlate in N/N^b^, M_1_/M_1_^b^, and N/N^b^ individuals, suggesting that these two regions may not be contiguous potentially due to gene conversions or other rearrangements.

In contrast, M_1_/M_1_^w^ patients had a higher frequency of multiple copies of *SMN2*, exon 7 (3 copies: 33.3%, 4 copies: 3.3%) when compared to M_1_/M_1_^b^ patients (3 copies: 6.7%, 4 copies: 4%), suggesting that gene conversion from telomeric *SMN1*, exon 7 to centromeric *SMN2*, exon 7 might be more common in M_1_/M_1_^w^ patients.

### Homozygous *SMN2*, Exons 7 and 8 Deletions Could Form Part of the Normal Variation in N/N^b^ Individuals

The high frequency of homozygous *SMN2*, exon 7 deletions in N/N^b^ individuals (27%) when compared to N/N^w^ individuals (6.7%) suggests that these deletions form part of the general variation in the black SA population. These frequencies are similar to international reports of ~10% (Corcia et al., 2002; Gamez et al., 2002).

**Figure 3:**
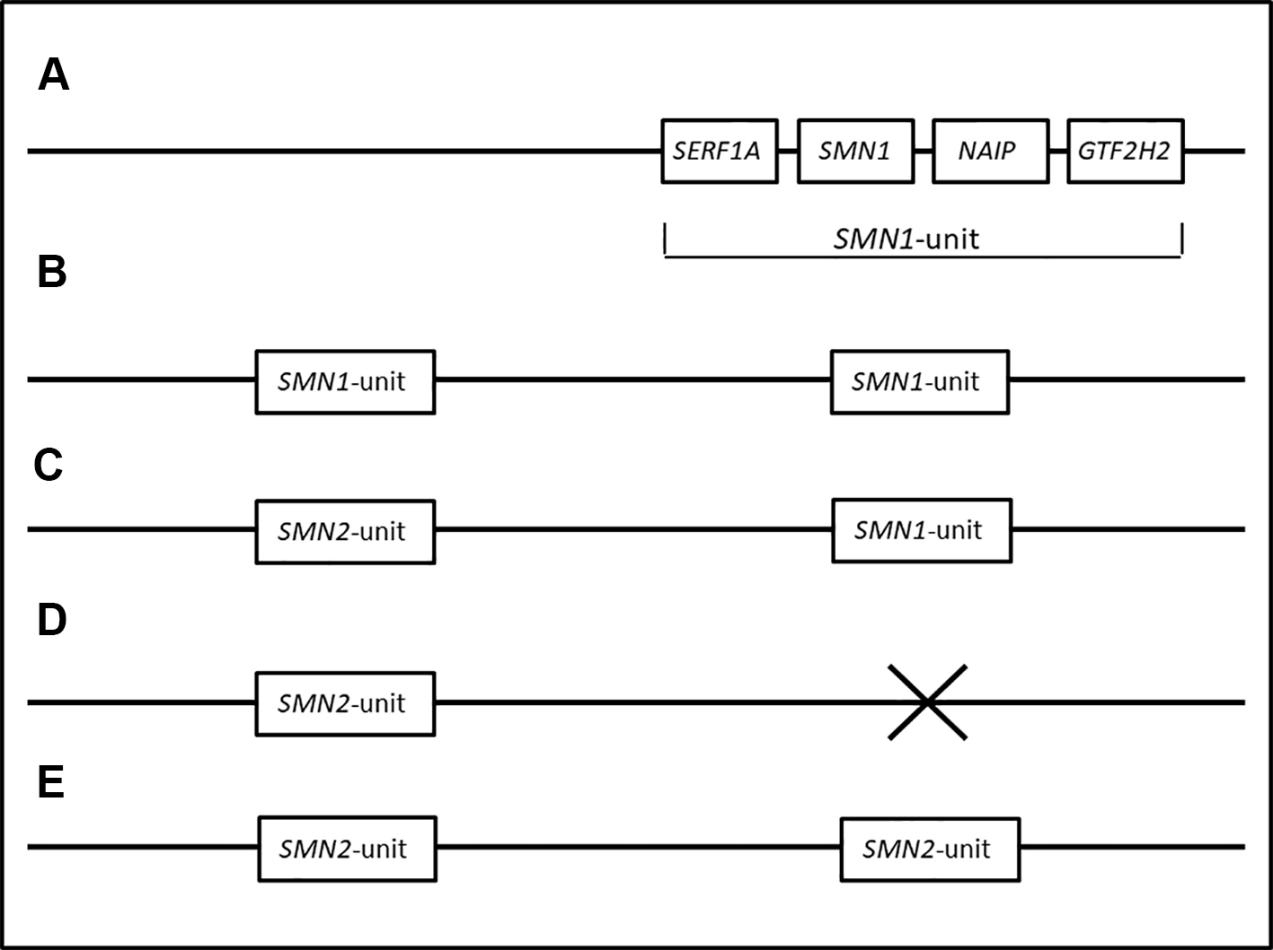
Hypothetical haplotypes representing the transformation of the SMN region from ancestral to modern populations. **(A)** represents the proposed order of genes in the *SMN1* region. The *SMN* region in primates and early humans are thought to have consisted of only one copy of the *SMN1* gene. **(B)** Duplications of the *SMN1* region resulted in multiple copies of the *SMN1* gene, frequently observed in individuals of African descent (Dennis et al., 2017). This is supported by the observation of multiple copies of *SMN1* in conjunction with the lack of *SMN2* in black SA individuals (N/N^b^ and U/U^b^ individuals). **(C)** Mutations in the duplicated *SMN1* gene resulted in the *SMN2* gene. A chromosome consisting of one *SMN1* and one *SMN2* gene.is thought to be the most common genotype seen in populations of European descent (N/N^w^ individuals). **(D, E)** represent individuals with a deletion of *SMN1*. A homozygous deletion of *SMN1*, exon 7 causes SMA (M_1_/M_1_^w^ and M_1_/M_1_^b^). **(E)** Deletions of *SMN1* in M_1_/M_1_^w^ individuals are commonly caused by gene conversions from *SMN1* to *SMN2*, resulting in multiple copies of *SMN2*.

Primates only have one copy of the *SMN1* gene. It has been hypothesized that the *SMN* region in early humans consisted of only the *SMN1* gene. Due to the hypervariable nature of the *SMN* region, duplications of the *SMN* region resulted in multiple copies of the *SMN1* gene, often observed in individuals of African descent (Dennis et al., 2017). This scenario is supported by the observation of multiple copies of *SMN1* in conjunction with the lack of *SMN2* in black SA individuals (N/N^b^ and U/U^b^ individuals). The duplicated *SMN1* gene diverged into the *SMN2* gene due to mutations (more specifically, the critical c.840C > T change in exon 7). A CNV containing both the *SMN1* and *SMN2* genes is more commonly observed in individuals of European descent (Kelter et al., 2000). A loss of *SMN1* could take place as a result of a deletion of *SMN1* or a gene conversion from *SMN1* to *SMN2*, an arrangement observed more frequently in individuals of European descent (van der Steege et al., 1996). The higher rate of gene conversion in white SA individuals supports this hypothesis (M_1_/M_1_^w^). **Figure 3** summarizes the different CNVs of the *SMN* region and their evolution.

Human-specific segmental duplication of the *SMN* region resulting in the inverted centromeric *SMN* duplication (including *SMN2*) has been estimated to have taken place 0.3 mya. The exact method of further duplication of the *SMN* ancestral structure to the structure of the human reference today has been difficult to determine due to polymorphic, palindromic duplications of the region (Dennis et al., 2017).

### No Novel Pathogenic CNVs Were Identified in U/U^b^ Patients

In contrast to previous SA reports of heterozygous *SMN1*, exon 7 deletions being present in 69.5% of U/U^b^ patients (Labrum et al., 2007), only 8.3% of U/U^b^ patients were confirmed to have heterozygous deletions of *SMN1*, exon 7 in this study. This discrepancy could firstly be due to MLPA analysis being a very robust technique, which has built-in statistical tests and extensive normalization, which are likely to yield more reliable and accurate results than the previously used in-house dosage assay (Labrum et al., 2007). In support of this hypothesis, seven individuals previously reported to have heterozygous deletions of *SMN1*, exon 7 on the in-house dosage assay were retested on MLPA of which four individuals had discrepant results. Two of these individuals had two copies and two individuals had three copies of *SMN1*, exon 7, which were mistaken for one copy on the in-house dosage assay.

Secondly the presence of multiple copies of *SMN1*, exon 7 could mask the actual heterozygous *SMN1*, exon 7 deletion rate in U/U^b^ patients.

No novel pathogenic CNVs were identified in U/U^b^ patients. The presence of potential large complex rearrangements in the black SA population not detectable by current standard diagnostic techniques is supported by the high variability and lack of correlation of copy number between different genes and exons seen in black SA individuals. *SMN* genes and exons may not have contiguous coding regions and the relationship between these complex rearrangements and the effect on SMN protein expression needs to be further investigated.

### Haplotype and CNV Pattern Analysis

As part of a previous study performed in the Division, linkage analysis, using two intragenic and six extragenic microsatellite markers across the *SMN1* gene, was performed to see if a common chromosomal background could be established in U/U^b^ patients. No clear haplotype or common allele was identified and it was reported that it was particularly difficult to construct haplotypes (Labrum et al., 2007).

Similarly, in this study, multiple gene and exon copies in the black SA population complicated haplotype construction. Only 44% of M_1_/M_1_^b^ families and 31.7% of N/N^b^ families were completely informative where the phase of the haplotype could be determined with certainty. The orientation of genes in the SMN region is not known and this study could not predict the arrangement of genes or exons even though the phase could be determined.

Potential novel events (sporadic deletions or duplications) were observed in 3.3% (2/61) of N/N^b^ families. A new mutation rate of 3.3% is not unexpected as novel mutations have been reported at a rate of 2% due to the high instability of this region (Wirth et al., 1997). Moosa and Dawood reported a paucity of family history in black SA families affected by SMA potentially due to SMA being more sporadic in this population (Moosa and Dawood, 1990) although this may be due to poor ascertainment. Sporadic mutations could be explained as a consequence of novel gene conversion and rearrangement events.

Two variants, c.885+83T > G and c.885+667delAT in exon 8 of the *SMN1* gene have been described to be associated with multiple *SMN1* copies on a single chromosome in combination with a *SMN1*, exon 7 deletion on the other chromosome in African American and Ashkenazi Jewish population groups. It was suggested that these two variants are associated with heterogous *SMN1* deletions (2:0) and could be useful in refining the carrier risk in individuals who have multiple copies of *SMN1*, exon 7 (Luo et al., 2014). The association of the c.885+83T > G and c.885+667delAT variants to heterozygous *SMN1* deletion (2:0) haplotypes in the black SA population was investigated as part of a previous unpublished study in the Division. Both the c.885+83T > G and c.885+667delAT variants were observed in 60% (3/5) of individuals with known heterozygous *SMN1* deletions (2:0) compared to 0% (0/7) of individuals known to have two copies of *SMN1*, exon 7 copy, one copy on each of their two chromosomes (1:1), suggesting that the two variants are associated with duplicated *SMN1*, exon 7 alleles. These two variants could be useful in refining the carrier risk of black SA individuals who have multiple copies of *SMN1*, exon 7.

### Implications for Diagnostic Testing

With the advent of new sequencing technologies, pan-ethnic population-based expanded carrier screening has been gaining momentum internationally. As an example, Israel has implemented a genetic screening program including carrier testing for SMA for couples of reproductive age (Zlotogora et al., 2016). The American College of Medical Genetics (ACMG) supports the inclusion of SMA into expanded carrier screening tests (Prior and Professional Practice and Guidelines Committee, 2008). Caution is advised against population screening in the black SA population, due to the presence of multiple copies of *SMN1*, exon 7 which could significantly impair accurate carrier detection and lead to false negative carrier results. MLPA may be useful in detecting the carrier risk in members of M_1_/M_1_^b^ families but it is highly recommended that MLPA results of samples referred for prenatal and carrier testing should be analyzed within a family context to identify the phase of multiple *SMN1* gene copies in all SA populations.

### Challenges and Limitations of the Study

The SMN region is extremely complex containing multiple pseudogenes (Selig et al., 1995) and repetitive sequences (Bürglen et al., 1996) within a large inverted segmental duplication. Due to this complexity, there is limited understanding of the exact order and location of genes in the SMN region. This is complicated even further since CNV trends observed in the various patient and control groups tested on MLPA, suggest that large rearrangements in the *SMN* region form part of the general variation within the black SA population.

It is well established that African populations have a higher level of genetic diversity than any other population (Tishkoff and Williams, 2002; Tishkoff and Kidd, 2004; Conrad et al., 2006; Pickrell et al., 2014). A local group of researchers who investigated CNVs in SA populations found that haplotype block lengths are significantly smaller in African populations when compared to non-African populations. These regions seem to coincide with recombination hotspots (Chimusa et al., 2015). Very few of these recombination hotspots seem to be shared between African and other populations (Choudhury et al., 2014; Chimusa et al., 2015). Perhaps the high variability of the *SMN* region in the black SA population could be due to frequent recombination events in the *SMN* region. This hypothesis is supported by a new mutation rate of 3.3% in this study which is comparable to the high new mutation rate seen in other populations (Wirth et al., 1997). Novel events may also influence the recurrence risk in black SA SMA families.

We need to identify and comprehend non-pathogenic CNVs in the general SA population to fully understand disease mechanisms overlaying these variations, specifically in the *SMN* region. The Southern African Human Genome Project (SAHGP) shows some promise in creating a better understanding of the baseline CNVs in the general black SA population (Pepper, 2011) although it is unlikely to provide detailed information on the architecture of the *SMN* region.

The high sequence homology of the *SMN1* and *SMN2* genes, with only a five nucleotide difference between the two genes and the highly variable CNVs of these genes make molecular diagnosis extremely challenging. This limitation complicates and restricts the design of primers and probes in this region and limits the choice of laboratory techniques that can be used to understand this region better. The P021-A2 MLPA kit was mainly designed to distinguish between exons 7 and 8 of the *SMN1* and *SMN2* gene, but cannot distinguish between exons 1, 4, and 6 of the *SMN1* and *SMN2* genes. This means that a combined result was observed for these probes. This makes it difficult to assign multiple copies of specific exons to either *SMN1* or *SMN2*. Similarly, the *NAIP/NAIPΨ*, exon 13 probe was designed to detect the combined copy number of the *NAIP* gene and its centromeric copy, *NAIPΨ* and the *GTF2H2*, exon 5 was designed to detect the combined copy number of telomeric-*GTF2H2* and centromeric-*GTF2H2*.

The copy numbers of *SMN1/2* exons 1, 4, 6, and 8 do not correlate fully with each other or with the *SMN1*, exons 7 and 8 copy numbers in any of the groups suggesting that CNVs of the *SMN1* and *SMN2* genes do not consist of complete gene copies and that the exons may be non-contiguous. This is further complicated by gene conversion events between *SMN1* and *SMN2*. Other factors which could potentially influence within- and between sample variance, is sample quality and experimental design. Result interpretation is therefore incredibly difficult and it is not possible to construct accurate CNV patterns using MLPA.

### Future Studies

MLPA testing cannot give us information about the functionality of potential multiple, partial copies. RNA expression studies may be able to quantify the expression of *FL-SMN* transcripts which may be a more accurate indication of the amount of SMN protein produced in U/U^b^ patients even in the presence of multiple *SMN1*, exon 7 copies. If multiple copies of the *SMN1* gene are present on MLPA, but there is no corresponding *FL-SMN* transcript, it could be indicative of partial/interrupted non-functional *SMN1* copies. The expression of SMN transcripts using real-time reverse transcription PCR (qRT-PCR) is being investigated as part of a current study in the Division.

Sixteen additional genes with overlapping phenotypes to SMA have been shown to be associated with non-5q forms of SMA (Peeters et al., 2014). Due to the lack of clinical information on patients referred for SMA testing to the Division it may be more practical to perform testing by an NGS neuromuscular panel first to exclude other related neuromuscular diseases and other causes of SMA before continuing SMA testing in individuals who test negative for the homozygous *SMN1*, exon 7 deletion.

A previous SA study sequenced the *SMN1* gene in patients found to have heterozygous *SMN1*, exon 7 deletions on the previously used in-house dosage assay to look for additional mutations in *SMN1* (Labrum et al., 2007). No pathogenic mutations were identified. Since the accuracy of the previously used in-house dosage assay has been questioned by this study, all U/U^b^ and M2/M2^b^ individuals who were found to have heterozygous deletions of SMN1, exon 7 should be sequenced to try and find a potential second pathogenic mutation. The high homology of the SMN1 and SMN2 genes complicates sequencing analysis however this challenge can be overcome with long range PCR targeting the *SMN1* gene, followed by nested PCR and Sanger sequencing of exons 1–8 (Kubo et al., 2015).

PacBio single molecule, real-time sequencing (SMRT) technology^6^ has shown some promise with resolving large CNVs. This technology is currently limited to 20 kb reads, which may still be too small to detect the full sequence of the *SMN* region, which is at least 500kb. The MinION (Oxford Nanoppore technologies) nanopore sequencer generate ultra-long sequencing reads of up to 800kb and have been shown to improve the accuracy and to close gaps in the reference human genome (Jain et al., 2018). These long range sequencing technologies could be investigated to try and further define the structure of the *SMN* region in the black SA population.

## Conclusion

This is the first report summarizing CNV patterns of the *SMN* region in African patients with known homozygous *SMN1* and *SMN2*, exon 7 deletions (M_1_/M_1_^b^, M_2_/M_2_^b^) and patients who have features clinically suggestive of SMA (U/U^b^). This is also the first report of CNVs patterns of the *SMN* region in the general black SA population.

Multiple copies of *SMN1*, exon 7 were observed as evidence of the marked hypervariability of the *SMN* region in the black SA population. These multiple copies potentially confound diagnostic and carrier testing and could potentially consist of partial, non-contiguous copies. Future studies investigating the expression of these multiple gene copies may provide information on their functional effect. No clear additional pathogenic CNV patterns were identified in U/U^b^ patients. This study emphasizes the lack of understanding of the architecture of the *SMN* region and the composition of CNVs in the black SA population. These factors need to be taken into account when counselling and performing diagnostic, carrier and prenatal testing in the black SA population.

## Data Availability Statement

All datasets generated for this study are included in the article and the supplementary files.

## Ethics Statement

This study was carried out in accordance with the recommendations of the 'WITS Medical Human Research Ethics Committee. Written informed consent of patients was not required as all DNA samples were referred and banked in a diagnostic setting and have been anonymised for the purpose of this study. The protocol was approved by the 'WITS Medical Human Research Ethics Committee (ethics clearance number: M130950).

## Author Contributions

EV, FE, and AK contributed to the conception and design of the study. JR assisted in identifying appropriate patients for this study. EV performed all laboratory work, MLPA analysis, haplotype analysis, and statistical analysis as part of her MSc (Medicine) Human Genetics degree (obtained with distinction). EV wrote the first draft of the manuscript. All authors contributed to manuscript revision, read and approved the submitted version.

## Funding

National Health Laboratory Service (NHLS) Research Trust Grant (2014-2DEV41-EVO1). WITS Faculty Research Council Grant.

## Conflict of Interest

The authors declare that the research was conducted in the absence of any commercial or financial relationships that could be construed as a potential conflict of interest.

## Footnotes

1 Online Mendelian Inheritance in Man (OMIM), OMIM accession numbers: 600354, 601627, http://www.omim.org.

2 MRC Holland MLPA general protocol and P021 product description, http://www.mlpa.com/WebForms/.

3 EMQN, https://www.emqn.org.

4 Real Statistics Using Excel, http://www.real-statistics.com.

5 Ensembl Genome Browser, https://www.ensembl.org

6 PacBio, https://www.pacb.com/smrt-science/smrt-sequencing/
